# Geographic variation of parathyroidectomy in patients receiving hemodialysis: a retrospective cohort analysis

**DOI:** 10.1186/s12893-016-0193-7

**Published:** 2016-11-29

**Authors:** James B. Wetmore, Jiannong Liu, Paul J. Dluzniewski, Areef Ishani, Geoffrey A. Block, Allan J. Collins

**Affiliations:** 1 Chronic Disease Research Group, Minneapolis Medical Research Foundation, 914 South 8th Street, Suite S4.100, Minneapolis, MN 55404 USA; 2 Division of Nephrology, Hennepin County Medical Center, Minneapolis, MN USA; 3 Department of Medicine, University of Minnesota, Minneapolis, MN USA; 4 Center for Observational Research, Amgen Inc, Thousand Oaks, CA USA; 5 Section of Renal Diseases and Hypertension, Minneapolis Veterans Administration Health Care System, Minneapolis, MN USA; 6 Denver Nephrology Clinical Research Division, Denver, CO USA

**Keywords:** End-stage renal disease, Dialysis, Mineral metabolism, Parathyroidectomy, Secondary hyperparathyroidism

## Abstract

**Background:**

Secondary hyperparathyroidism (SHPT) is associated with adverse outcomes in patients receiving maintenance dialysis. Parathyroidectomy is a treatment for SHPT; whether parathyroidectomy utilization varies geographically in the US is unknown.

**Methods:**

A retrospective cohort analysis was undertaken to identify all patients aged 18 years or older who were receiving in-center hemodialysis between 2007 and 2009, were covered by Medicare Parts A and B, and had been receiving hemodialysis for at least 1 year. Parathyroidectomy was identified from inpatient claims using relevant International Classification of Diseases, Ninth Revision, Clinical Modification procedure codes. Patient characteristics and End-Stage Renal Disease Network (a proxy for geography) were ascertained. Adjusted odds ratios for parathyroidectomy were estimated from a logistic model.

**Results:**

A total of 286,569 patients satisfied inclusion criteria, of whom 4435 (1.5%) underwent PTX. After adjustment for a variety of patient characteristics, there was a 2-fold difference in adjusted odds of parathyroidectomy between the most- and least-frequently performing regions. Adjusted odds ratios were more than 20% higher than average in four networks, and more than 20% lower in four networks.

**Conclusions:**

Parathyroidectomy use varies substantially by geography in the US; the factors responsible should be further investigated.

## Background

Secondary hyperparathyroidism (SHPT) is associated with adverse outcomes in patients receiving maintenance dialysis [1, 2]. Anecdotally, physicians appear to have widely variable criteria regarding which patients they choose to refer for parathyroidectomy, at least in the US. Perhaps reflecting uncertainty over its role, rates of parathyroidectomy have changed substantially over time in recent decades [3]. While guidelines recommend parathyroidectomy in patients with severe SHPT [4], how it might be used most optimally is uncertain. Parathyroidectomy has been shown to be associated with improved outcomes in some studies [5, 6]; however, it has also been shown to be associated with mortality, protracted hypocalcemia, and over-suppression of parathyroid hormone (PTH) [7], and its results with regard to mineral metabolic control are often suboptimal [8]. Thus, understanding the differences between hemodialysis patients who do and do not undergo parathyroidectomy may be important. However, the effect of geographic variation, which is associated with a variety of outcomes and care differences in the dialysis population [9, 10] has not been examined in the context of parathyroidectomy. We therefore conducted a retrospective cohort study to examine whether parathyroidectomy use varies geographically in the United States.

## Methods

Using the United States Renal Data System end-stage renal disease database, we identified patients aged 18 years or older who were receiving in-center hemodialysis between 2007 and 2009, were covered by Medicare Part A (inpatient, outpatient, skilled nursing facility, hospice, or home health agency) and Part B (physician/supplier) as primary payer, and had been receiving hemodialysis for at least 1 year. Parathyroidectomy was identified from inpatient claims using International Classification of Diseases, Ninth Revision, Clinical Modification procedure codes 06.81 (complete parathyroidectomy), 06.89 (partial parathyroidectomy and parathyroidectomy not otherwise specified), and 06.95 (parathyroid tissue reimplantation).

Patient characteristics, derived from the end-stage renal disease database Medical Evidence Report and Medicare claims, were assessed on the parathyroidectomy date and on January 1 for non-parathyroidectomy patients. Characteristics included age, sex, race, body mass index, cause of renal disease, dialysis duration, and common comorbid conditions, as have been used previously [11]. Our proxy for geography was US End-Stage Renal Disease Network (*n* = 18, Table 1), geographically based regions designed to facilitate care and monitor quality on a regional level. Adjusted odds ratios (ORs) and 95% confidence intervals (CIs) for parathyroidectomy were estimated from a logistic model adjusting for the factors described above. The adjusted ORs for the renal networks were calculated using the whole nation as the reference. All statistical analyses were conducted using SAS software, Version 9.2, SAS Institute Inc., Cary, NC, USA.

**Table 1:** End-stage renal disease networks and associate US states

Network number	States and territories
1	Connecticut, Maine, Massachusetts, New Hampshire, Rhode Island, Vermont
2	New York
3	New Jersey, Puerto Rico, Virgin Islands
4	Delaware, Pennsylvania
5	District of Columbia, Maryland, Virginia, West Virginia
6	Georgia, North Carolina, South Carolina
7	Florida
8	Alabama, Mississippi, Tennessee
9	Indiana, Kentucky, Ohio
10	Illinois
11	Michigan, Minnesota, North Dakota, South Dakota, Wisconsin
12	Iowa, Kansas, Missouri, Nebraska
13	Arkansas, Louisiana, Oklahoma
14	Texas
15	Arizona, Colorado, Nevada, New Mexico, Utah, Wyoming
16	Alaska, Idaho, Montana, Oregon, Washington
17	American Samoa, Guam, Mariana Islands, Hawaii, Northern California
18	Southern California

## Results

We identified 286,569 patients who satisfied our inclusion criteria, of whom 4435 (1.5%) underwent parathyroidectomy (Table 2). Parathyroidectomy frequency was 2.3 fold greater, in unadjusted terms, for the least-frequently performing region (0.97% of patients, Network 18) compared with the most-frequently performing region (2.20% of patients, Network 6).

**Table 2:** Characteristics of patients who did and did not undergo parathyroidectomy

	PTX		Non-PTX	
	*n*	%	*n*	%
Total	4435	100	282,134	100
Age at PTX, years				
19–44	1764	39.8	38,830	13.8
45–64	2154	48.6	110,788	39.3
65–74	410	9.2	69,550	24.7
≥ 75	107	2.4	62,966	22.3
Race				
White	1685	38.0	156,638	55.5
Black	2551	57.5	108,246	38.4
Other	199	4.5	17,250	6.1
Sex				
Male	2298	51.8	155,257	55.0
Female	2137	48.2	126,877	45.0
ESRD primary cause				
Diabetes	1013	22.8	128,202	45.4
Hypertension	1462	33.0	81,231	28.8
Glomerulonephritis	934	21.1	27,250	9.7
Other/unknown/missing	1026	23.1	45,451	16.1
BMI, kg/m^2^				
< 18	151	3.4	8074	2.9
18– < 25	1217	27.4	88,838	31.5
25– < 30	1026	23.1	78,938	28.0
30– < 35	772	17.4	49,310	17.5
35– < 40	517	11.7	25,987	9.2
≥ 40	536	12.1	23,383	8.3
Missing	216	4.9	7604	2.7
Dialysis duration, years				
1– < 3	538	12.1	151,778	53.8
3– < 5	970	21.9	58,341	20.7
> 5	2927	66.0	72,015	25.5
Comorbidities				
Diabetes	1954	44.1	185,029	65.6
ASHD	1542	34.8	131,678	46.7
CHF	1973	44.5	144,792	51.3
CVA/TIA	522	11.8	57,532	20.4
PVD	1389	31.3	112,972	40.0
Dysrhythmia	1057	23.8	77,320	27.4
Other cardiac disease	1623	36.6	91,955	32.6
Network				
1	151	3.4	9407	3.3
2	192	4.3	16,808	6.0
3	156	3.5	11,887	4.2
4	118	2.7	11,689	4.1
5	260	5.9	17,080	6.1
6	667	15.0	29,663	10.5
7	253	5.7	16,076	5.7
8	364	8.2	17,060	6.1
9	293	6.6	21,152	7.5
10	154	3.5	11,977	4.3
11	252	5.7	18,814	6.7
12	199	4.5	11,188	4.0
13	254	5.7	12,073	4.3
14	487	11.0	27,892	9.9
15	182	4.1	12,016	4.3
16	139	3.1	7254	2.6
17	136	3.1	11,860	4.2
18	178	4.0	18,238	6.5

*ASHD* atherosclerotic heart disease, *BMI* body mass index, *CHF* congestive heart failure, *CVA/TIA* cerebrovascular accident/transient ischemic attack, *ESRD* end-stage renal disease, *PTX* parathyroidectomy, *PVD* peripheral vascular disease

**Fig. 1:**
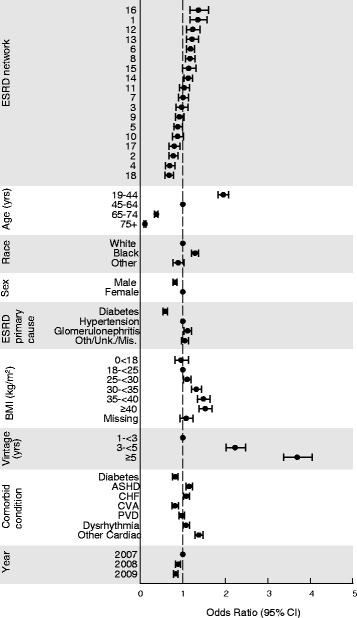
Odds ratios for factors associated with parathyroidectomy. ASHD, atherosclerotic heart disease; CHF, congestive heart failure; CVA, cerebrovascular accident; ESRD, end-stage renal disease; PVD, peripheral vascular disease

Network was associated with substantial variability in likelihood of parathyroidectomy (Fig. 1). Even after adjustment for all characteristics in Table 2, adjusted ORs for parathyroidectomy varied from 0.67 (95% CIs 0.58–0.78) to 1.37 (1.17–1.60) between the least- and most-frequently performing regions. Adjusted ORs were more than 20% higher than the national level in four networks and more than 20% lower in four networks.

In addition, younger age (adjusted OR 1.95, 95% CI 1.83–2.08, vs. age 45–64 years), female sex (1.23, 1.16–1.30), black race (1.29, 1.21–1.37 vs. white), dialysis duration > 5 years (3.70, 3.27–4.05 vs. 1- < 3 years), and atherosclerotic heart disease (1.15, 1.07–1.23) were associated with parathyroidectomy (*P* < 0.001). Diabetes (0.82, 0.76–0.89) and history of stroke (0.82, 0.74–0.89) were inversely associated with parathyroidectomy.

Results for the multivariable model for factors associated with parathyroidectomy are shown in Table 3.

**Table 3:** Multivariable model for factors associated with parathyroidectomy

Factors	HR (95% CI)	*P*
Age at PTX, years		
19–44	1.95 (1.83–2.08)	<0.001
45–64	1 (Referent)	
65–74	0.37 (0.34–0.42)	< 0.001
≥ 75	0.11 (0.09–0.13)	< 0.001
Race		
White	1 (Referent)	
Black	1.29 (1.21–1.37)	< 0.001
Other	0.89 (0.77–1.03)	0.11
Sex		
Male	0.82 (0.77–0.87)	< 0.001
Female	1 (Referent)	
ESRD primary cause		
Diabetes	0.58 (0.53–0.64)	< 0.001
Hypertension	1 (Referent)	
Glomerulonephritis	1.11 (1.02–1.21)	0.011
Other/unknown/missing	1.04 (0.96–1.13)	0.30
BMI, kg/m^2^		
< 18	0.96 (0.82–1.13)	0.62
18– < 25	1 (Referent)	
25– < 30	1.10 (1.02–1.20)	0.016
30– < 35	1.32 (1.21–1.44)	< 0.001
35– < 40	1.48 (1.34–1.64)	< 0.001
≥ 40	1.53 (1.38–1.69)	< 0.001
Missing	1.08 (0.94–1.24)	0.29
Dialysis duration, years		
1– < 3	1 (Referent)	
3– < 5	2.23 (2.02–2.47)	< 0.001
≥ 5	3.70 (3.37–4.05)	< 0.001
Comorbid conditions		
Diabetes	0.82 (0.76–0.89)	< 0.001
ASHD	1.15 (1.07–1.23)	< 0.001
CHF	1.08 (1.01–1.15)	0.019
CVA/TIA	0.82 (0.74–0.89)	< 0.001
PVD	0.97 (0.91–1.04)	0.42
Dysrhythmia	1.08 (1.00–1.16)	0.058
Other cardiac disease	1.37 (1.29–1.47)	< 0.001
ESRD Network		
16	1.37 (1.17–1.60)	< 0.001
1	1.35 (1.17–1.57)	< 0.001
12	1.24 (1.09–1.40)	0.001
13	1.24 (1.08–1.37)	0.001
6	1.18 (1.09–1.28)	< 0.001
8	1.17 (1.06–1.29)	0.002
15	1.14 (0.99–1.31)	0.067
14	1.13 (1.03–1.23)	0.008
11	1.03 (0.92–1.16)	0.60
7	1.01 (0.90–1.13)	0.91
3	0.97 (0.84–1.12)	0.69
9	0.92 (0.83–1.02)	0.12
5	0.88 (0.79–0.99)	0.032
10	0.88 (0.76–1.01)	0.070
17	0.80 (0.68–0.93)	0.005
2	0.78 (0.68–0.88)	< 0.001
4	0.69 (0.59–0.82)	< 0.001
18	0.67 (0.58–0.78)	< 0.001
Year		
2007	1 (Referent)	
2008	0.89 (0.83–0.95)	0.001
2009	0.83 (0.78–0.89)	< 0.001

*ASHD* atherosclerotic heart disease, *BMI* body mass index, *CHF* congestive heart failure, *CI* confidence interval, *CVA/TIA* cerebrovascular accident/transient ischemic attack, *ESRD* end-stage renal disease, *HR* hazard ratio, *PTX* parathyroidectomy, *PVD* peripheral vascular disease

## Discussion

SHPT treatment presents a complex clinical challenge. Practice guidelines provide direction [4] but suffer from lack of randomized clinical trial data, resulting in uncertainty about the benefits and risks of parathyroidectomy. Understanding use of parathyroidectomy is important, given widely varying recent data demonstrating both clinical benefits [5, 6], as well as high rates of adverse events and suboptimal mineral metabolic outcomes [7, 8]. Our large retrospective analysis demonstrated substantial geographic variation in parathyroidectomy use. This difference was not driven solely by outliers at the extremes; AORs were 20% higher or lower than unity in eight Networks. This could reflect regional differences in many potential factors, including provider-related ones such as particular treatment approaches instilled during training, access to qualified parathyroid surgeons, or local “cultures” of treatment, all of which might play substantial roles in how care is differentially rendered [12].

Certain demographic factors, specifically younger age and black race, were also associated with likelihood of parathyroidectomy; this was not unexpected given that both of these factors have been previously reported to be associated with more severe SHPT [2, 13]. Dialysis duration was also associated with parathyroidectomy, possibly because the changes that characterize severe parathyroid gland dysregulation may take many years to develop [14]; alternatively, providers may be resorting to parathyroidectomy only after prolonged attempts at other interventions prove fruitless. The inverse associations between older age and history of stroke and parathyroidectomy may reflect poor surgical candidacy in the provider’s estimation.

Our study was limited by lack of patient-level data about degree of PTH control, SHPT therapies employed, or other SHPT markers such as serum calcium and phosphorus, which likely predict the parathyroidectomy decision. For example, use of cinacalcet, which has been shown to reduce rates of parathyroidectomy [15], might vary widely by region, although we have no *a priori* reason to posit this and it seems unlikely to account for a more than 2-fold variation in parathyroidectomy rates. Additionally, we lack information about geographic variation in renal transplant; fewer individuals in areas in which early transplant occurs more commonly might be at risk of developing severe SHPT and subsequently undergoing parathyroidectomy. Again, given the magnitude of variation between the most- and least-frequently parathyroidectomy performing regions, case mix alone is unlikely to fully account for it.

## Conclusion

Even after adjustment of a variety of case-mix variables, use of parathyroidectomy varies substantially by geography in the US; the factors responsible should be further investigated. Given recent information about the potential risks associated with parathyroidectomy [7, 8], the factors responsible for shaping the decision to undertake it should also be the subject of future investigation.

## Abbreviations

CI: Confidence interval
OR: Odds ratio
PTH: Parathyroid hormone
SHPT: Secondary hyperparathyroidism
